# *Ad Libitum* Feeding in Broiler Breeder Hens Alters the Transcriptome of Granulosa Cells of Pre-Hierarchal Follicles

**DOI:** 10.3390/ani11092706

**Published:** 2021-09-16

**Authors:** Laurie Francoeur, Claire S. Stephens, Patricia A. Johnson

**Affiliations:** Department of Animal Science, College of Agriculture and Life Sciences, Cornell University, Ithaca, NY 14853, USA; lf389@cornell.edu (L.F.); ces277@cornell.edu (C.S.S.)

**Keywords:** broiler breeder, nutrition and reproduction, egg production, follicle development, granulosa cell, RNA sequencing

## Abstract

**Simple Summary:**

Broiler breeds of chickens have been bred for fast growth and feed efficiency, while laying breeds have been bred for optimal egg production. As a consequence of intense selective breeding in broiler breeds, egg production is greatly reduced and leads to inefficient reproduction. One strategy used by producers to improve egg production in broiler breeds has been to limit feed allowance. In this study, we aimed to identify differences in ovarian gene expression between broiler breeder hens fed high (*ad libitum*) and low feed allowances. Several differences in gene expression were identified, which may explain the decreased egg production seen in broiler breeder hens fed *ad libitum*. These results inform the poultry industry on the biology of broiler breeder hens fed *ad libitum* and how levels of high feed intake affect reproductive efficiency.

**Abstract:**

Intense selective breeding of chickens has resulted in suboptimal egg production in broiler breeder hens. This reproductive phenotype is exacerbated by *ad libitum* feeding, which leads to excessive and disorganized follicular growth. One strategy used to improve broiler breeder hens’ reproductive efficiency is restricted feeding. In this study, we sought to identify transcriptional changes, which translate the level of dietary intake into increased follicle selection. Broiler breeder hens (*n* = 16 per group) were raised according to commercial guidelines until 28 weeks of age and then randomly assigned to an *ad libitum* diet (FF) or continued on a restricted diet (RF) for 6 weeks. Following dietary treatment, FF hens (*n* = 2) with excessive follicle selection and RF hens (*n* = 3) with normal follicle selection were selected for RNA-sequencing. Transcriptomes of granulosa cells from 6–8-mm follicles were sequenced to identify transcriptional differences in the follicle population from which selection was made for the preovulatory stage. Differential expression analysis identified several genes known to play a role in follicle development (CYP11A1, STAR, INHA, and INHBB) that are upregulated in FF hens. These changes in gene expression suggest earlier granulosa cell differentiation and steroidogenic competency in the granulosa layer from FF hens.

## 1. Introduction

Broiler chickens are selected for fast growth and feed efficiency, and laying hens are selected for optimal egg production. Selection pressure for ideal production traits has resulted in vastly different reproductive efficiencies between broiler breeders and laying hens. Although laying hens can lay almost one egg per day, broiler hens have aberrant follicle growth which often leads to erratic laying, multiple ovulations, and poor-quality eggs [1]. These events contribute to suboptimal reproduction in these hens. One strategy used to improve broiler breeder hens’ reproductive efficiency is restricted feeding, which results in an ovarian phenotype more like that of the laying hen. While restricting feed (RF) results in improved egg production compared to *ad libitum* feeding (FF), dietary change alone is not sufficient to reach the egg-laying efficiency seen in layer breeds. At the ovarian follicle level, FF broiler breeder hens have multiple hierarchies and significantly more preovulatory follicles than hens on a restricted diet [2,3]. Despite excessive follicle development, ovulation is erratic, resulting in low egg production. It is not known how feeding level directly impacts follicle selection and growth.

Follicle selection occurs when one follicle from a pool of growing follicles begins to become dominant and continues maturation until it ovulates [4]. In the hen, follicle selection is characterized by two important events: granulosa cell differentiation and the initiation of progesterone synthesis. Prior to selection, granulosa cells are said to be undifferentiated and steroidogenically incompetent [5]. Undifferentiated granulosa cells express low levels of the steroidogenic acute regulatory protein (STAR), luteinizing hormone receptor (LHR), cytochrome P450scc (CYP11A1), and follicle stimulating hormone receptor (FSHR) [6,7] (reviewed in [8]). At the time of selection, granulosa cells undergo transcriptional changes in key regulators and receptors. STAR and CYP11A1 expression increases [6,9] and follicles begin to produce progesterone [10], with the largest preovulatory follicle producing the highest amount of progesterone and stimulating the LH surge for ovulation [11,12]. In a reproductively efficient hen, the largest preovulatory follicle will ovulate each day and one 6–8-mm follicle will be selected to replace it and replenish the preovulatory follicle pool (reviewed in [4,8]). In the FF broiler breeder hen, there is a large number of unorganized preovulatory follicles [2], which contributes to the reproductive inefficiency of these birds. Although factors associated with follicle selection in laying hens have been identified [4,6,9], differences in pre-hierarchal follicle transcriptomes have not yet been studied in broiler breeder hens in response to dietary treatment.

In this study, we investigated transcriptional changes in granulosa cells of 6–8-mm follicles to identify factors that may be disrupting normal follicle selection in FF broiler breeder hens. Specifically, we hypothesized that the increased feed intake in these hens disrupts important regulators of follicle development, resulting in increased follicle numbers.

## 2. Materials and Methods

### 2.1. Animals

One-day-old broiler breeder chicks (Cobb 700, *n* = 32) were donated by Cobb-Vantress and raised in floor pens in Cornell University’s Poultry Facility according to commercial guidelines [13]. Birds were kept on a light cycle of 15 h of light and 9 h of dark. At 28 weeks of age, hens were randomly assigned to one of two pens and fed either an *ad libitum* diet (FF, *n* = 16) or were continued on a restricted feed diet of 146 g/day/bird (RF, *n* = 16) for an additional 6 weeks according to commercial guidelines [13] and as previously described in Stephens and Johnson, 2017 [14]. Throughout the length of the experiment, egg production per pen was measured daily. During the sixth week of the dietary treatment, hens were weighed, euthanized using CO_2_, and samples were collected. Egg production was calculated as eggs/hen/days in a one-week period. Egg production during weeks 1–5 are reported as these represented full weeks for both treatment groups. All animal procedures were approved by the Institutional Animal Care and Use Committee of Cornell University (protocol number 2009-0036).

### 2.2. Sample Collection

The liver, fat pad, and ovary were removed from the hen and weighed. Organ weights were normalized to the body weight. Upon collection, the ovary was placed in ice cold Krebs-Ringer bicarbonate buffer and follicles were collected and separated by size. Follicles from the 3–5-mm, 6–8-mm, and >9-mm size categories were counted and 6–8-mm follicles were removed. Granulosa cells were collected and pooled following the procedure outlined in Wang et al. [15] and stored in RLT lysis buffer at −80 °C until further processing.

### 2.3. RNA Extraction

Total RNA was extracted from granulosa cells using an RNeasy Mini kit with optional on-column DNase treatment (Qiagen Inc., Valencia, CA, USA). The quantity and purity of the samples were analyzed using spectrophotometry (Implen, Munich, Germany). RNA integrity was then determined by a Fragment Analyzer (Advanced Analytical, Ames, IA, USA). All samples had a RQN of >9.9.

### 2.4. RNA Sequencing and Quality Control

Samples were selected for RNA-sequencing based on the number of preovulatory follicles (>9-mm). For the FF group (*n* = 3), hens with more than 10 preovulatory follicles were selected to represent an excessive follicle selection phenotype. For the RF group (*n* = 3), hens with 6–7 preovulatory follicles were selected to represent a normal follicle selection phenotype. The granulosa cell layer from 6–8-mm follicles of these two phenotypes was collected as described above and used for RNA sequencing.

RNA samples were submitted to Cornell’s Transcriptional Regulation and Expression Facility for cDNA library preparation. Samples were enriched by PolyA+ RNA isolation using the NEBNext Poly(A) mRNA Magnetic Isolation Module (New England Biolabs, Ipswich, MA, USA). Libraries were then generated using the NEBNext Ultra II [Directional] RNA Library Prep Kit (New England Biolabs, Ipswich, MA, USA). Before sequencing, libraries were quantified using a Qubit 2.0 (dsDNA HS kit; Thermo Fisher, Waltham, MA, USA). cDNA libraries were sequenced on Illumina’s NextSeq500 (Illumina, San Diego, CA, USA) at a depth of 75 bp for a minimum of 31M reads per sample. FastQ files were first processed through trim-galore (Barbraham Institute, Cambridge, UK) as a quality control step to trim adaptors and filter for low quality reads. The sample files were then aligned to the Galgal6 genome using the RNA-seq aligner STAR [16]. A minimum of 91.7% reads were mapped to the genome in each sample (Table S1).

To verify for sample clustering by biological replicates, hclust in R was used [17]. This analysis identified one outlier in the FF group which was discarded in further analyses for a final sample of *n* = 2 in this group. A principle component analysis (PCA) was utilized to visualize the variance among samples.

### 2.5. RNA Sequencing Analysis

Differential expression analysis was conducted to identify differentially expressed genes (DEGs) between FF and RF hens using DeSEQ2. Criteria for DEGs were a false discovery rate (FDR) of <0.05, a log fold change of >1, and a minimum read count of 200. Gene ontology enrichment analysis with an FDR cutoff of 0.05 using ShinyGo v0.61 [18] was conducted on DEGs upregulated in FF and RF hens and generated the ten most significant terms. Additionally, Qiagen’s Ingenuity Pathway Analysis (IPA) was used to identify the predicted upstream regulators [19,20] of DEGs.

### 2.6. cDNA Synthesis and Real-Time qPCR

Gene expression of select DEGs (CYP11A1, STAR, INHA, and INHBB) was quantified using real-time qPCR to validate RNA-sequencing results. Total RNA from granulosa cells was extracted as described above. One µg of total RNA from each sample was reverse transcribed to cDNA in a 20-µL reaction using the High-Capacity cDNA Reverse Transcription kit (Applied Biosystems, Foster City, CA, USA).

The AB StepOnePlus Real-Time PCR System (Applied Biosystems, Foster City, CA, USA) was used for quantitative PCR. Each sample was analyzed in a 25-µL volume reaction with a final concentration of 1X for the Power SYBR Green (Applied Biosystems, Foster City, CA, USA) and 300 nM for the primers. Each reaction consisted of 1 µL of cDNA and samples, standards, no template controls, and reactions lacking reverse transcriptase were assessed in duplicates. Sample expression for each gene was determined from the standard curve and normalized to 18S expression. To measure the housekeeping gene, 18S primers from Ambion^®^ QuantumRNA™ 18S Internal Standard (ThermoFisher Scientific, Waltham, MA, USA) were used. Previously published primer sequences were used for CYP11A1 and STAR [21]. Primers for INHA and INHBB were designed to span exon-exon junctions using Primer-BLAST [22]. Primer sequences for gene targets can be found in Table 1.

### 2.7. Statistical Analysis

All body parameters and mRNA expression data were compared between the RF and FF groups using PROC GLM of the SAS 9.4 software (SAS Institute Inc., Cary, NC, USA). Week was included as a fixed effect in the generalized linear model for egg production.

## 3. Results

### 3.1. Body Parameters and Ovarian Morphology

Following 6 weeks of dietary treatment, FF hens had significantly higher live body weights than RF hens (*p* < 0.001, Table 2). Both fat pad and liver weights were also significantly higher in FF hens compared to RF hens when normalized to body weight (*p* < 0.001, Table 2).

**Figure 1:**
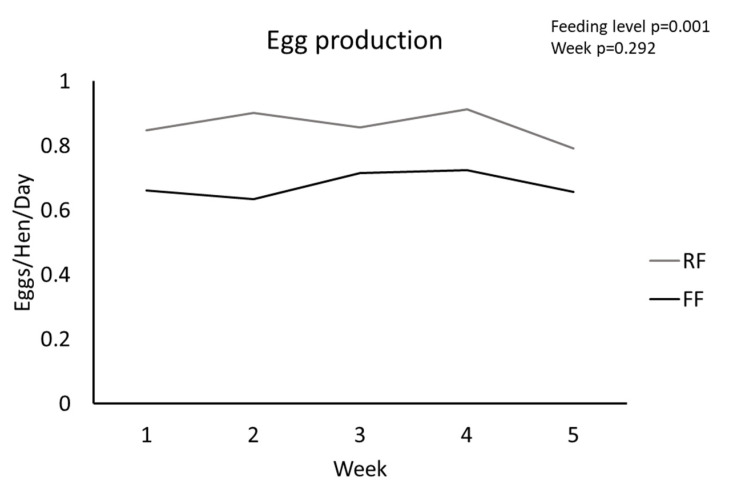
Egg production (eggs/hen/day) of RF and FF hens in response to dietary treatment (*n* = 16 per group, *p* = 0.013). *p*-values for individual fixed effects are in the top right corner.

FF hens had significantly more follicles greater than 9-mm in diameter compared to RF hens (*p* = 0.002, Table 2), indicating an increase in the number of follicles selected into the preovulatory hierarchy. Ovarian weight normalized to body weight was not different between RF and FF hens (*p* = 0.101, Table 2). Egg production was significantly higher for RF hens than FF hens throughout the course of the experiment (*p* = 0.013, Table 2 and Figure 1).

### 3.2. RNA Sequencing and Differential Gene Expression

**Figure 2:**
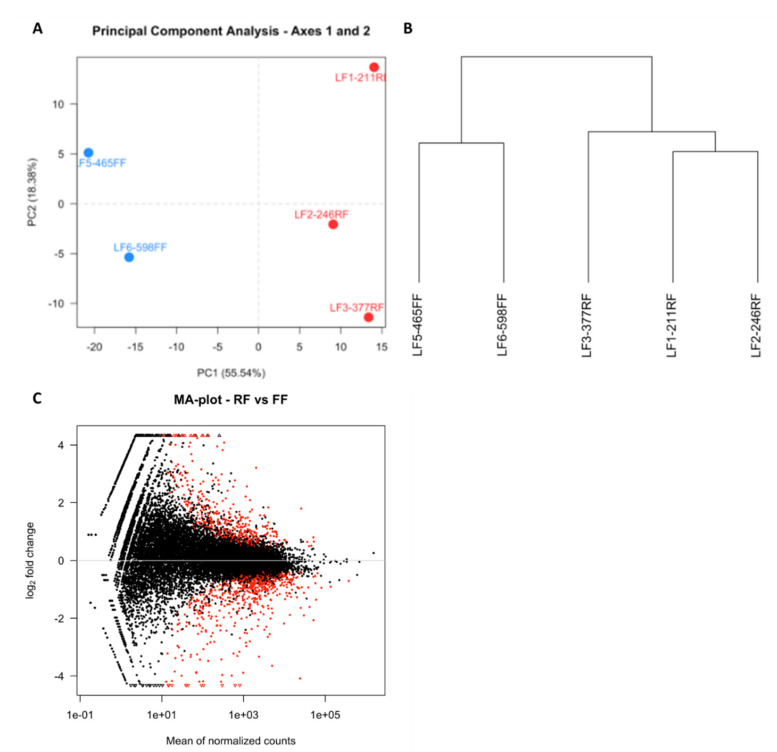
Principle component analysis, hierarchal clustering, and MA plot for RNA-sequencing data of granulosa cells from 6–8-mm follicles from RF and FF hens. (**A**) Principle component analysis comparing FF (*n* = 2, blue) and RF (*n* = 3, red) transcriptomes. (**B**) Hierarchal clustering of FF (*n* = 2) and RF (*n* = 3) samples. (**C**) MA plot comparing normalized counts in FF and RF hens. Black dots represent expressed genes and red dots indicate DEGs. Genes located below the x-axis represent genes more highly expressed in FF hens and genes located above the x-axis represent genes more highly expressed in RF hens.

**Figure 3:**
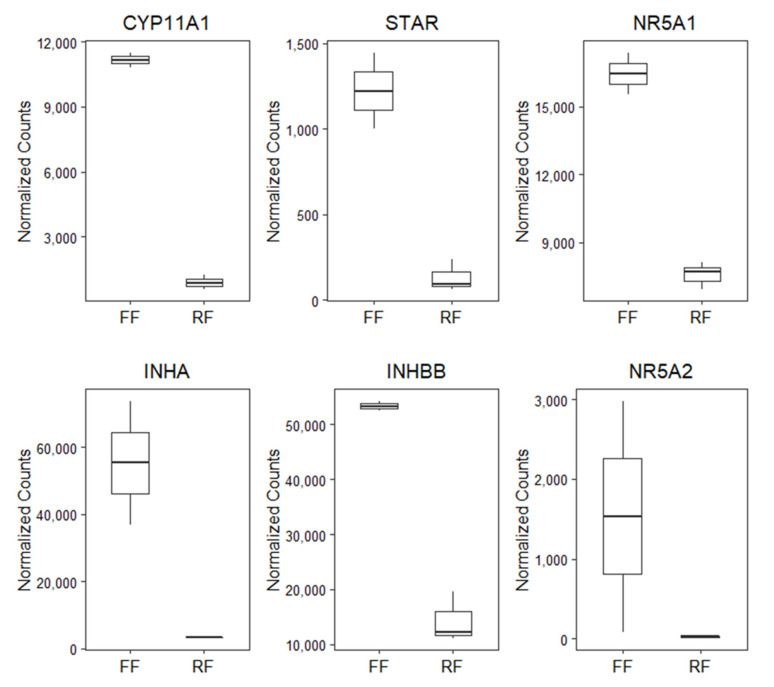
Boxplots of normalized counts from RNA-seq analysis comparing FF (*n* = 2) and RF (*n* = 3) gene expression of select DEGs (CYP11A1, STAR, NR5A1, INHA, INHBB, and NR5A2).

**Figure 4:**
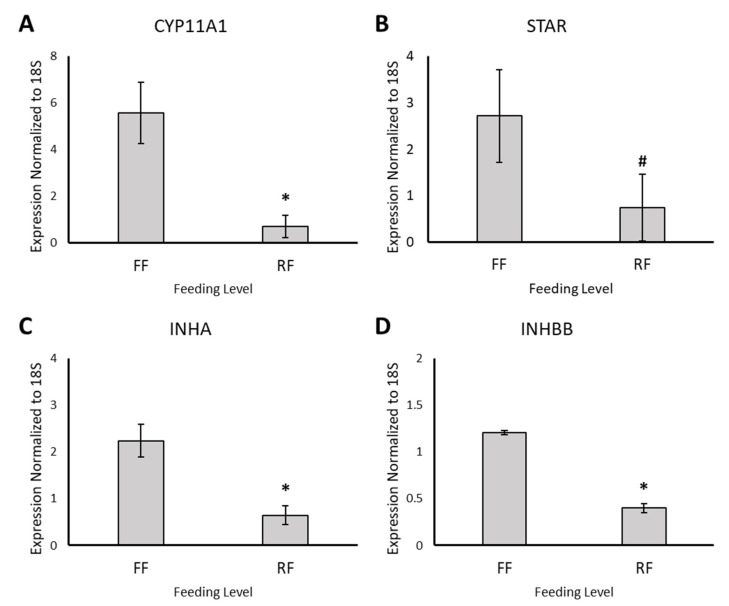
CYP11A1, STAR, INHA, and INHBB mRNA expression in granulosa cells of 6–8-mm follicles in FF and RF hens. Bars represent the mean expression normalized to 18S ± SD in granulosa cells of 6–8-mm follicles in FF (*n* = 2) and RF hens (*n* = 3) (*p* > 0.01, *; *p* = 0.08, **#**). (**A**) CYP11A1 mRNA expression (*p* < 0.01); (**B**) STAR mRNA expression (*p* = 0.08); (**C**) INHA mRNA expression (*p* < 0.01); and (**D**) INHBB mRNA expression (*p* < 0.01).

PCA revealed that samples clustered with biological replicates along the first component (Figure 2A) and this was confirmed by hierarchal clustering (Figure 2B). Differential gene expression analysis resulted in 350 DEGs, of which 207 and 143 were upregulated in FF hens and RF hens, respectively (Figure 2C). The top 50 upregulated genes in FF and RF hens are listed in Table 3 and Table 4, respectively. Of the genes upregulated in the granulosa cells of 6–8-mm follicles of FF hens, several are known to be important for steroidogenesis (CYP11A1, STAR, NR5A1, and NR5A2) and in regulating FSH (INHA and INHBB) (Figure 3). CYP11A1, STAR, INHA, and INHBB mRNA expression were measured using qPCR to validate RNA-sequencing results. CYP11A1, INHA, and INHBB expression was significantly higher (*p* < 0.01) and STAR expression tended to be higher in granulosa cells of 6–8-mm follicles of FF hens (*p* = 0.08, Figure 4).

### 3.3. Enrichment Analysis

Enrichment analysis categorized DEGs by functional category. The top ten significant terms associated with DEGs upregulated in FF and RF hens are listed in Table 5 and Table 6, respectively. Several DEGs upregulated in FF hens are associated with terms related to lipid metabolism such as “lipid catabolic process” and “cellular lipid catabolic process” (Table 5). The term “transmembrane transport” categorized the most upregulated DEGs in FF hens (Table 5). Other terms of note reflect cellular reorganization in the granulosa cells of 6–8-mm follicles in FF hens including “positive regulation of actin filament bundle assembly” and “positive regulation of cellular component biogenesis” (Table 5). DEGs upregulated in RF hens are associated with cellular homeostasis. Several of the top functional terms are associated with ion transport: “ion transport”, “cation transport”, “metal ion transport”, and “ion transmembrane transport” (Table 6). Other terms are associated with maintaining cell physiology, such as “regulation of system process” and “positive regulation of cell size” (Table 6).

### 3.4. Upstream Regulators

**Figure 5:**
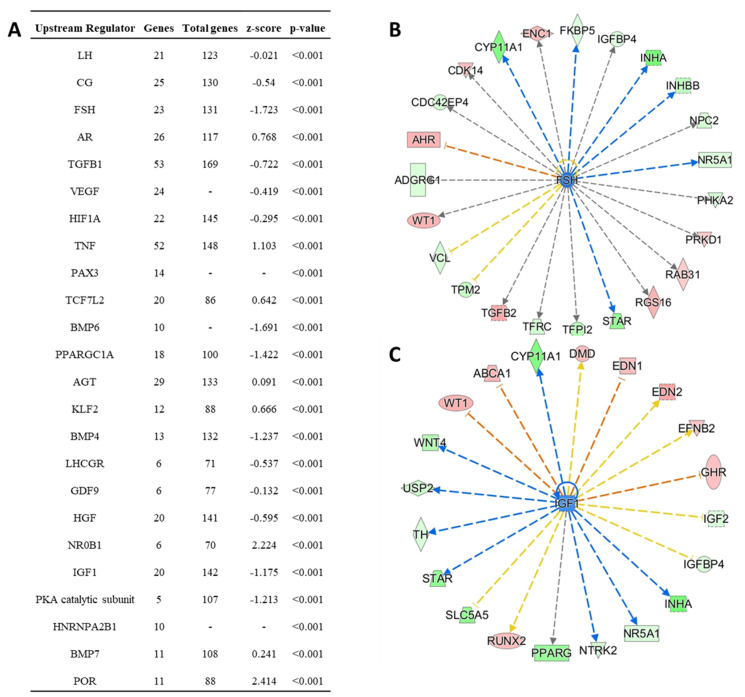
Upstream regulator analysis of differentially expressed genes in RF and FF hens. (**A**) The top 25 predicted upstream regulators of DEGs from the granulosa cells of 6–8-mm follicles from FF and RF hens are shown. The upstream regulator, number of gene targets in the DEG dataset, total number of genes found in the IPA network for the specific regulator, z-score of activation, and *p*-value of overlap are indicated. (**B**) FSH network wheel of gene targets in dataset. Green shapes are upregulated and red shapes are downregulated in FF hens (z-score = −1.723). Line colors indicate the state of activation (blue = activation; orange = inhibition; yellow = findings inconsistent with downstream gene state; and grey = unpredicted effects). (**C**) IGF1 network wheel of gene targets in dataset (z-score = −1.175). Colors of the shapes and lines have the same meaning as in panel (**B**).

Using IPA’s upstream regulator analysis, several transcriptional regulators were predicted to be upstream of DEGs. Among them are gonadotropins (LH, CG, and FSH), and growth factors (TGFB1, BMP6, AGT, BMP4, GDF9, HGF, IGF1, and BMP7) (Figure 5A). Two predicted upstream regulators of note are FSH with 23 downstream DEGs (Figure 5B) and Insulin-like Growth Factor 1 (IGF1) with 20 downstream DEGs (Figure 5C). Of the DEGs, seven genes (CYP11A1, EDN, IGFBP4, INHA, NR5A, STAR, and WT1) are predicted by IPA to be regulated by both FSH and IGF1.

## 4. Discussion

Broiler breeder hens fed *ad libitum* have increased follicle selection and excessive follicular growth, which results in decreased egg production. Commercial producers can increase egg production by restricting the dietary intake in these hens, resulting in a more regulated follicle hierarchy. In this study, we sought to generate hypotheses for the increased follicle development observed in FF hens by identifying differences in the transcriptomes of granulosa cells from 6–8-mm follicles, the stage of follicle selection.

Consistent with a previous study in our lab, we found that the preovulatory follicle number was significantly higher in FF hens when compared to RF hens [23]. Although we have previously found a significantly higher number of 3–5-mm and 6–8-mm follicles in FF hens compared to RF hens [23], we did not observe this in the present study. Another group using a similar experimental protocol also indicated no difference of numbers in these follicle size categories [24]. Our finding of a higher number of preovulatory follicles in FF hens is consistent with the hypothesis of increased follicle selection in response to increased dietary intake. At the time of follicle selection, granulosa cells become differentiated and gain the capacity to produce progesterone [25]. In the laying hen, one 6–8-mm follicle is selected approximately each day. This follicle enters the preovulatory stage to replace the recently ovulated follicle and this permits both an organized follicle hierarchy and efficient egg production. Given the increased number of preovulatory follicles observed in FF hens, the process of follicle selection is likely increased. We found transcriptional changes in the granulosa cell layer of 6–8-mm follicles, the stage at which follicle selection occurs.

In laying hens, the transcriptomes of granulosa cells from a 6-mm follicle and those from the most recently selected preovulatory follicle (F5) have been sequenced to investigate the transcriptional changes during the transition from unselected to selected follicles [26]. Of the top 50 DEGs identified in the granulosa cells of the recently selected follicle of the laying hen, 22 overlap with those we identified in the granulosa cells (6–8-mm follicles) of the FF hen. Among the notable genes in common between these two groups are CYP11A1 and STAR. CYP11A1 and STAR are important for the production of progesterone, an important functional change acquired at the time of follicle selection [10]. Given the strong overlap in gene expression between these two populations, it is possible that granulosa cells of 6–8-mm follicles of FF hens are more differentiated than those of RF hens. This early differentiation may occur in the granulosa layers of multiple 6–8-mm follicles, thereby disrupting the follicle hierarchy.

In the laying hen, granulosa cells begin to produce progesterone from cholesterol following selection into the preovulatory follicle stage [10]. This steroidogenic competency is associated with an increase in STAR and CYP11A1 expression [6,9]. Given the increased expression of STAR and CYP11A1 in granulosa cells from 6–8-mm follicles of the FF hen, progesterone production may increase earlier in follicle development than in laying hens or RF hens. Furthermore, two transcriptional activators, namely NR5A1 (synonym SF-1) and NR5A2 (synonym LRH-1), were found to be upregulated in the FF hen. NR5A1 binds to the promotor of CYP11A1 to increase its transcription and the production of progesterone in the granulosa cells of rats [27]. A second transcription factor, NR5A2, also increases CYP11A1-promoter activity in human granulosa cells [28] and in the presence of FSH, increases CYP11A1 mRNA and progesterone synthesis in cultured rat granulosa cells [29]. The increased STAR, CYP11A1, NR5A1, and NR5A2 expression in granulosa cells of 6–8-mm follicles of FF hens suggests that the production of progesterone may be initiated at this stage of follicle development, earlier than observed in laying hens. It has previously been proposed that production of progesterone may be initiated earlier in follicle development in FF hens [30]. Both the F1 and F2 follicles in FF hens have been shown to secrete higher progesterone in FF hens compared to RF hens, where only the F1 follicle secretes high progesterone levels [30]. Alternatively, these hens may be gaining the machinery for steroidogenesis during the 6–8-mm follicle stage and producing higher levels of progesterone once they enter the preovulatory stage. Dysregulation in the production of progesterone may be stimulating multiple ovulations and therefore be contributing to the double ovulations that are often observed in FF hens.

In addition to changes in the ovary, we found that full-feeding increased body weight, liver weight/BW, and fat pad weight/BW, as we have previously shown [23]. Increased dietary intake results in increased adiposity, with FF hens showing increased fat pad weight and increased plasma triglyceride and cholesterol levels compared to RF hens [23]. Enrichment analysis showed “lipid catabolic process” and “cellular lipid catabolic process” as some of the most significant terms for granulosa cells of 6–8-mm follicles from FF hens. This suggests that granulosa cells of 6–8-mm follicles may adjust their physiology to accommodate the increased amount of plasma lipids. Interestingly, the DEG with the highest fold change in both the granulosa cells of the most recently selected follicle of the laying hen [26] and the granulosa cells of 6–8-mm from FF broiler breeder hens is Carboxyl Ester Lipase (CEL). CEL is known to be excreted from the pancreas and has effects on lipid absorption in the intestine (reviewed in [31]). Among its roles, CEL functions in cholesterol absorption, in the reverse transport to the liver, and potentially in the cholesterol uptake by cells (reviewed in [31]). In the chicken, CEL is mainly expressed in the pancreas [32]. CEL expression in the pancreas has been shown to be influenced by dietary cholesterol in rats [33]; however, to our knowledge, our study is the first demonstrating that diet influences CEL expression in gonads. Although the role of CEL has not yet been investigated in the ovary, it may facilitate cholesterol transport in the cell. Two other lipid transporters, namely ABCA12 and SLC25A1, a mitochondrial transporter important for downstream endogenous cholesterol synthesis (summarized in [34]), are also increased in granulosa cells of 6–8-mm follicles of FF hens. Upregulation of CEL, ABCA12, and SLC25A1 may be increasing cholesterol availability to granulosa cells of 6–8-mm follicles in FF hens with higher levels of plasma cholesterol and facilitating enhanced progesterone production.

Although dietary intake affects the transcriptomes of granulosa cells of 6–8-mm follicles, the factors directly contributing to these changes remain unclear. The upstream regulator analysis predicted FSH and IGF1 to have effects on several DEGs upregulated in FF hens. In the hen, granulosa cells become responsive to FSH at the time of selection and one of the 6–8-mm follicles shows an increase in FSHR mRNA [7]. FSH increases cAMP levels to stimulate STAR and CYP11A1 transcription [6,9]. In the current study, INHBB, INHA, STAR, and CYP11A1 were upregulated in granulosa cells of 6–8-mm follicles from FF hens and predicted to be downstream of FSH. Reported plasma FSH levels in RF and FF hens are variable as some studies found no difference [2], elevated levels [35,36,37], or decreased FSH plasma levels in FF compared to RF hens [24]. More research is needed to determine the effect of increased dietary intake on FSH plasma levels.

In a previous study, we investigated liver transcriptome differences between FF and RF hens and showed that FF hens have elevated liver IGF1 mRNA and protein compared to RF hens [23]. At the preovulatory follicle stage, IGF1 can increase progesterone production and expression of STAR, CYP11A1, and 3βHSD in chicken granulosa cells [38]. In granulosa cells of pre-hierarchal follicles, IGF1 increases cell proliferation [39]. In mammals, FSH and IGF1 have been shown to increase CYP11A1 and STAR expression synergistically [40,41,42,43]. Using upstream regulator analysis, we found seven genes (CYP11A1, EDN, IGFBP4, INHA, NR5A, STAR, and WT1) downstream of both FSH and IGF1. These genes could be targets for synergistic effects of FSH and IGF1 in the hen, and elevated IGF1 in FF hens [23] may synergize with FSH in FF hens to enhance follicle development.

## 5. Conclusions

This is the first study to investigate differences in the transcriptome of pre-hierarchal follicles between broiler breeder hens fed at different levels. Three hundred and fifty genes were found to be differentially expressed between FF and RF hens in granulosa cells from 6–8-mm follicles. Several genes involved in follicle selection were upregulated in pre-hierarchal follicles of FF hens, suggesting an ovarian effect of dietary treatment at early stages in follicle development. Findings from this study suggest that granulosa cells of 6–8-mm follicles may mature earlier in FF hens than in RF hens, particularly with respect to capacity for progesterone synthesis. In addition, increased plasma cholesterol levels, FSH, and IGF1 may be involved in some of these transcriptional changes. These hypotheses have opened new research avenues which we are actively pursuing. Ultimately, this research helps clarify the processes contributing to the reproductive inefficiencies observed in broiler breeder hens. A greater understanding of the etiology of these reproductive inefficiencies can provide targets for treatment and genetic selection to improve the reproductive health and welfare of these hens.

## Figures and Tables

**Table 1:** Primer Sequences for RT-qPCR.

Target Gene	Primer Sequence
CYP11A1	F 5′-ACTTCAAGGGACTGAGCTTTGGGT-3′ R 5′ AGTTCTCCAGGATGTGCATGAGGA 3′
STAR	F 5′-TGCCTGAGCAGCAGGGATTTATCA- 3′ R 5′- TGGTTGATGATGGTCTTTGGCAGC-3′
INHA	F 5′-TCTTCCCTTCCACAGACGTG- 3′ R 5′- CTGTAGAACCAGAGCTGGGC-3′
INHBB	F 5′-TTCGCCGAGACAGACGAT- 3′ R 5′- TTACTTTTCGCCTGCTGCCT-3′

**Table 2:** Mean body weight, normalized fat pad, liver, and ovary weights, as well as follicle numbers (3–5-mm, 6–8-mm, and follicles >9-mm) and egg production (eggs/hen/day) for RF and FF hens in response to dietary treatment (*n* = 15–16 per group). Means are presented as means ± SD.

Parameter	RF	FF	*p*-Value
Body weight (g)	3389 ± 162	4483 ± 359	<0.0001
Fat pad weight/BW	0.015 ± 0.007	0.030 ± 0.008	<0.0001
Liver weight/BW	0.028 ± 0.005	0.048 ± 0.010	<0.0001
Ovary weight/BW	0.018 ± 0.003	0.021 ± 0.005	0.101
3–5-mm follicles	32.2 ± 11.0	36.3 ± 12.0	0.332
6–8-mm follicles	11.6 ± 3.9	10.7 ± 3.7	0.519
Follicles > 9-mm	6.3 ± 0.9	8.2 ± 2.0	0.002
Eggs/hen/day	0.862 ± 0.048	0.678 ± 0.039	0.013

**Table 3:** Top 50 upregulated differentially expressed genes in FF hens, including the Ensembl gene ID, gene name, log_2_(FC), and FDR, and average normalized read counts for FF and RF groups are indicated for each gene.

GeneID	Gene Name	log_2_(FC)	FDR	FF Average	RF Average
ENSGALG00000034436	CEL	−8.6	0.01	749	2
ENSGALG00000002182	NR5A2	−6.4	<0.01	1531	18
ENSGALG00000038884	SRL	−4.9	<0.01	250	8
ENSGALG00000045327	-	−4.9	<0.01	222	8
ENSGALG00000026808	TMEM72	−4.8	<0.01	1922	70
ENSGALG00000006440	-	−4.2	<0.01	652	35
ENSGALG00000050830	SV2A	−4.2	<0.01	237	13
ENSGALG00000054770	INHA	−4.1	<0.01	55,417	3271
ENSGALG00000010269	KCNAB1	−4.0	<0.01	1174	75
ENSGALG00000042836	KCNH2	−4.0	<0.01	688	44
ENSGALG00000047771	PGF	−3.9	<0.01	2220	150
ENSGALG00000034982	CYP11A1	−3.6	<0.01	11,165	893
ENSGALG00000029968	GADD45B	−3.5	<0.01	300	27
ENSGALG00000001207	PLCH2	−3.4	0.01	630	59
ENSGALG00000006598	SORL1	−3.3	<0.01	5205	517
ENSGALG00000041932	SLC5A5	−3.3	<0.01	1534	155
ENSGALG00000003242	STAR	−3.2	<0.01	1222	132
ENSGALG00000010364	AADAC	−3.2	0.01	1694	189
ENSGALG00000040355	TCF24	−3.1	<0.01	220	26
ENSGALG00000004974	PPARG	−3.0	<0.01	2454	314
ENSGALG00000005884	MAPKKK3L	−2.9	<0.01	270	37
ENSGALG00000011242	OBSL1	−2.8	<0.01	2359	328
ENSGALG00000042607	RSPO3	−2.8	<0.01	321	45
ENSGALG00000014938	ABHD3	−2.8	<0.01	396	56
ENSGALG00000038399	PLEKHA6	−2.8	<0.01	2921	420
ENSGALG00000050611	-	−2.7	<0.01	1650	257
ENSGALG00000041143	UMOD	−2.6	<0.01	7916	1266
ENSGALG00000011537	PDE10A	−2.6	<0.01	325	53
ENSGALG00000008815	LRRN4	−2.5	<0.01	8678	1484
ENSGALG00000011608	INF2	−2.5	<0.01	2394	418
ENSGALG00000011803	EMP1	−2.5	<0.01	347	61
ENSGALG00000019077	-	−2.5	<0.01	2109	372
ENSGALG00000043234	HBA1	−2.5	<0.01	236	43
ENSGALG00000042929	-	−2.4	<0.01	746	139
ENSGALG00000010326	FLVCR2	−2.4	<0.01	530	98
ENSGALG00000006453	TF	−2.4	<0.01	232	43
ENSGALG00000037603	SESN2	−2.4	<0.01	6298	1195
ENSGALG00000003750	PLCG1	−2.4	<0.01	3056	590
ENSGALG00000033683	PHOSPHO1	−2.3	<0.01	6924	1408
ENSGALG00000016415	MAP7D2	−2.3	<0.01	4248	882
ENSGALG00000044649	P2RX2	−2.3	<0.01	1134	235
ENSGALG00000016954	RGCC	−2.3	<0.01	416	87
ENSGALG00000008537	EPHB3	−2.3	<0.01	516	108
ENSGALG00000036728	PRDM16	−2.2	<0.01	436	92
ENSGALG00000041708	WNT4	−2.2	<0.01	18,324	4040
ENSGALG00000014734	-	−2.2	<0.01	1166	260
ENSGALG00000018803	-	−2.1	<0.01	415	98
ENSGALG00000009512	TFPI2	−2.1	<0.01	94,942	22,674
ENSGALG00000049157	DOK4	−2.0	<0.01	4274	1034
ENSGALG00000038458	LOXL1	−2.0	<0.01	1624	401

**Table 4:** Top 50 upregulated differentially expressed genes in RF hens, including the Ensembl gene ID, gene name, log_2_(FC) and FDR, and average normalized read counts for FF and RF groups are indicated for each gene.

GeneID	Gene Name	log_2_(FC)	FDR	FF Average	RF Average
ENSGALG00000051980	-	6.5	0.01	2	229
ENSGALG00000036798	COL4A1	4.1	0.02	32	535
ENSGALG00000007819	PDZRN3	4.0	<0.01	24	361
ENSGALG00000015908	COL12A1	3.8	<0.01	34	491
ENSGALG00000043754	GLUL	3.2	<0.01	340	3158
ENSGALG00000010858	LRP2	3.0	0.03	56	446
ENSGALG00000040755	ANGPT4	2.9	<0.01	44	320
ENSGALG00000048104	-	2.8	<0.01	54	367
ENSGALG00000003670	MAFB	2.8	<0.01	137	938
ENSGALG00000016843	COL4A2	2.8	0.01	40	271
ENSGALG00000033338	GPT2	2.6	<0.01	60	374
ENSGALG00000030065	TENM3	2.6	<0.01	306	1805
ENSGALG00000009405	GRIA2	2.6	<0.01	143	839
ENSGALG00000034453	SAMD11	2.4	<0.01	113	604
ENSGALG00000050840	APCDD1	2.2	<0.01	355	1641
ENSGALG00000006172	ABCC8	2.1	0.01	120	534
ENSGALG00000038364	NOV	2.1	<0.01	492	2083
ENSGALG00000016820	GAS6	2.1	<0.01	49	207
ENSGALG00000013697	CNDP1	2.1	<0.01	106	443
ENSGALG00000011200	THBS2	2.0	<0.01	65	264
ENSGALG00000014178	-	2.0	<0.01	82	318
ENSGALG00000011623	ADAMTS3	1.9	<0.01	208	803
ENSGALG00000031916	ZP1	1.8	0.01	78	283
ENSGALG00000000667	EDN2	1.8	<0.01	1580	5639
ENSGALG00000026055	PALM	1.8	<0.01	60	210
ENSGALG00000009687	KCNK2	1.8	<0.01	10,362	35,972
ENSGALG00000004812	FAM129A	1.8	0.02	58	203
ENSGALG00000007268	-	1.8	<0.01	192	669
ENSGALG00000002671	-	1.8	<0.01	660	2230
ENSGALG00000032836	-	1.7	<0.01	137	445
ENSGALG00000012595	AGTPBP1	1.7	<0.01	96	310
ENSGALG00000001768	TENM2	1.7	0.01	340	1095
ENSGALG00000011994	SYNPO2	1.7	0.02	66	215
ENSGALG00000036883	MET	1.7	<0.01	619	1988
ENSGALG00000009612	TGFB2	1.7	<0.01	188	600
ENSGALG00000045776	CPN2	1.7	0.01	90	282
ENSGALG00000002081	MMP28	1.6	<0.01	112	348
ENSGALG00000015542	PLPPR1	1.6	<0.01	105	324
ENSGALG00000017065	-	1.6	0.05	84	259
ENSGALG00000011145	TRIL	1.6	0.01	99	301
ENSGALG00000034085	-	1.6	0.01	207	627
ENSGALG00000031534	ARID5B	1.6	<0.01	360	1081
ENSGALG00000026981	NHSL1	1.6	0.01	370	1102
ENSGALG00000027514	-	1.6	<0.01	1168	3445
ENSGALG00000016251	-	1.6	<0.01	1485	4369
ENSGALG00000012115	WT1	1.5	<0.01	1514	4420
ENSGALG00000010902	CERS6	1.5	0.05	153	448
ENSGALG00000051001	-	1.5	0.01	294	844
ENSGALG00000009495	FGFR2	1.5	<0.01	229	648
ENSGALG00000012834	AKR1D1	1.5	<0.01	460	1297

**Table 5:** Enrichment analysis of upregulated differentially expressed genes in FF hens. This table shows the top ten significant terms associated with DEGs found to be upregulated in FF granulosa cells of 6–8-mm follicles. The functional category, number of genes found in the DEG list, total number of genes found in the database for the specific functional category term, and the FDR are listed.

Functional Category	Genes	Total Genes	FDR
Lipid catabolic process	10	186	2.17 × 10^−4^
Heme export	2	2	1.65 × 10^−2^
Positive regulation of actin filament bundle assembly	4	44	3.44 × 10^−2^
Cellular lipid catabolic process	6	126	3.44 × 10^−2^
Proteoglycan biosynthetic process	4	48	3.81 × 10^−2^
Inositol trisphosphate biosynthetic process	3	21	3.81 × 10^−2^
Positive regulation of cellular component biogenesis	9	345	3.81 × 10^−2^
Heme transport	2	6	4.43 × 10^−2^
Inositol trisphosphate metabolic process	3	24	4.43 × 10^−2^
Transmembrane transport	17	1122	4.43 × 10^−2^

**Table 6:** Enrichment analysis of upregulated differentially expressed genes in RF hens. This table shows ten significant terms associated with DEGs found to be upregulated in RF granulosa cells of 6–8-mm follicles. The functional category, number of genes in the DEG list, total number of genes in the database for the specific functional category term, and the FDR are listed.

Functional Category	Genes	Total Genes	FDR
Ion transport	12	1117	3.26 × 10^−2^
Cation transport	10	757	3.26 × 10^−2^
Muscle contraction	5	171	3.26 × 10^−2^
Nitric oxide mediated signal transduction	2	11	3.26 × 10^−2^
Regulation of heart contraction	4	112	3.26 × 10^−2^
Regulation of nitric oxide mediated signal transduction	2	6	3.26 × 10^−2^
Metal ion transport	8	545	3.26 × 10^−2^
Ion transmembrane transport	10	790	3.26 × 10^−2^
Regulation of system process	6	291	3.26 × 10^−2^
Positive regulation of cell size	2	6	3.26 × 10^−2^

## Data Availability

The data presented in this study are openly available on NCBI’s Gene Expression Omnibus (Edgar et al., 2002) and are accessible through GEO Series accession number GSE175887 (https://www.ncbi.nlm.nih.gov/geo/query/acc.cgi?acc=GSE175887).

## Supplementary Materials

The following are available online at https://www.mdpi.com/article/10.3390/ani11092706/s1, Table S1: RNA-Seq Alignment Summary.
