# Supplementary material for: Ad Libitum Feeding in Broiler Breeder Hens Alters the Transcriptome of Granulosa Cells of Pre-Hierarchal Follicles
Source: Animals (Basel). 2021 Sep 16;11(9):2706. doi: 10.3390/ani11092706 (PMC8472788; doi:10.3390/ani11092706)
Supplement: Supplementary file 1 [file animals-11-02706-s001.zip › animals-1337493-supplementary.pdf]

Supplementary Materials

Table S1: RNA-Seq Alignment Summary. Summary of mapped reads to the Galgal6 genome assembly.

cDNA Library	Total Reads	Mapped Reads	% Aligned
211RF	31302807	28332671	90.5%
246RF	39972051	36131559	90.4%
377RF	49634581	45080210	90.8%
465FF	36996330	33194095	89.7%
598FF	45763471	40732555	89.0%
